# Application of machine learning in risk stratification of obesity-related osteoarthritis: A study based on a nationally representative sample

**DOI:** 10.1515/jtim-2025-0060

**Published:** 2025-11-25

**Authors:** Qingzhu Wu, A Liang, Yihao Tian, Hongqiu Li, Zhen Liu, Kebin Xu, Baizhou Liang, Yudong Wu, Fu Ren, Xin Li

**Affiliations:** Department of Sports Medicine Orthopaedics, Central Hospital Affiliated to Shenyang Medical College, Shenyang, Liaoning Province, China; Department of Pathology, General Hospital of Northern Theater Command, Beifang Hospital of China Medical University, Shenyang, Liaoning Province, China; General Surgery Department of the First Affiliated Hospital of Jinzhou Medical University, Jinzhou, Liaoning Province, China; School of Public Health, Shenyang Medical College, Shenyang, Liaoning Province, China; Key Laboratory of Human Ethnic Specificity and Phenomics of Critical Illness, Shenyang, Liaoning Province, China; Key Laboratory of Phenomics in Shenyang, Shenyang, Liaoning Province, China; Shenyang Key Laboratory of Prevention and Treatment of Systemic Important Diseases Associated with Oral Diseases, Shenyang, Liaoning Province, China; The Affiliated Stomatological Hospital of China Medical University, Shenyang, Liaoning Province, China; School of Stomatology, Shenyang Medical College, Shenyang, Liaoning Province, China; School of Basic Medicine, Shenyang Medical College, Shenyang, Liaoning Province, China

## To the editor

Osteoarthritis (OA), a degenerative joint disease characterized by chronic pain and functional impairment.^[1]^ Global Burden of Disease (GBD) data demonstrate a significant association between high body mass index (BMI) and OA incidence.^[2]^ Although the underlying mechanisms may involve increased biomechanical stress and systemic inflammation, OA development among individuals with obesity exhibits notable heterogeneity.^[3]^ Therefore, BMI serves as an imperfect risk predictor, underscoring the need to identify a more granular set of predictive factors to account for this heterogeneity This study employed machine learning (ML) to analyze national survey data and identify other key OA predictors in adults with obesity.

This study utilized data from the 2005-2023 NHANES cycle.^[4]^ NHANES is a nationally representative program designed to evaluate the health and nutritional status of adults and children in the United States. The inclusion criteria were as follows: (1) aged 20 years or older; (2) diagnosed with obesity, defined as having a BMI ≥ 30 kg/m^2^; (3) available data on OA status (the primary outcome); and (4) available data on the predictor variables included in the analysis. Participants with missing data on outcome variables or BMI were excluded. After screening, a total of 9495 adults with obesity were included in the study, among whom 3036 (32.0%) had OA. This study also utilized data from the GBD Study 2021 to examine the burden and temporal trends of OA attributable to BMI. For this analysis, we extracted annual estimates of disability-adjusted life years (DALYs) for knee and hip OA attributed to high BMI. Meanwhile, we explored the predictive performance of nine ML algorithms (K-nearest neighbor [KNN], decision tree [DT], elastic net [ENET], LightGBM, logistic regression, multilayer perceptron [MLP], random forest [RF], radial support vector machine [SVM], and XGBoost). Furthermore, we utilized the SHapley Additive exPlanations method to interpret the models, identify key predictive factor.^[5]^

**Figure 1:**
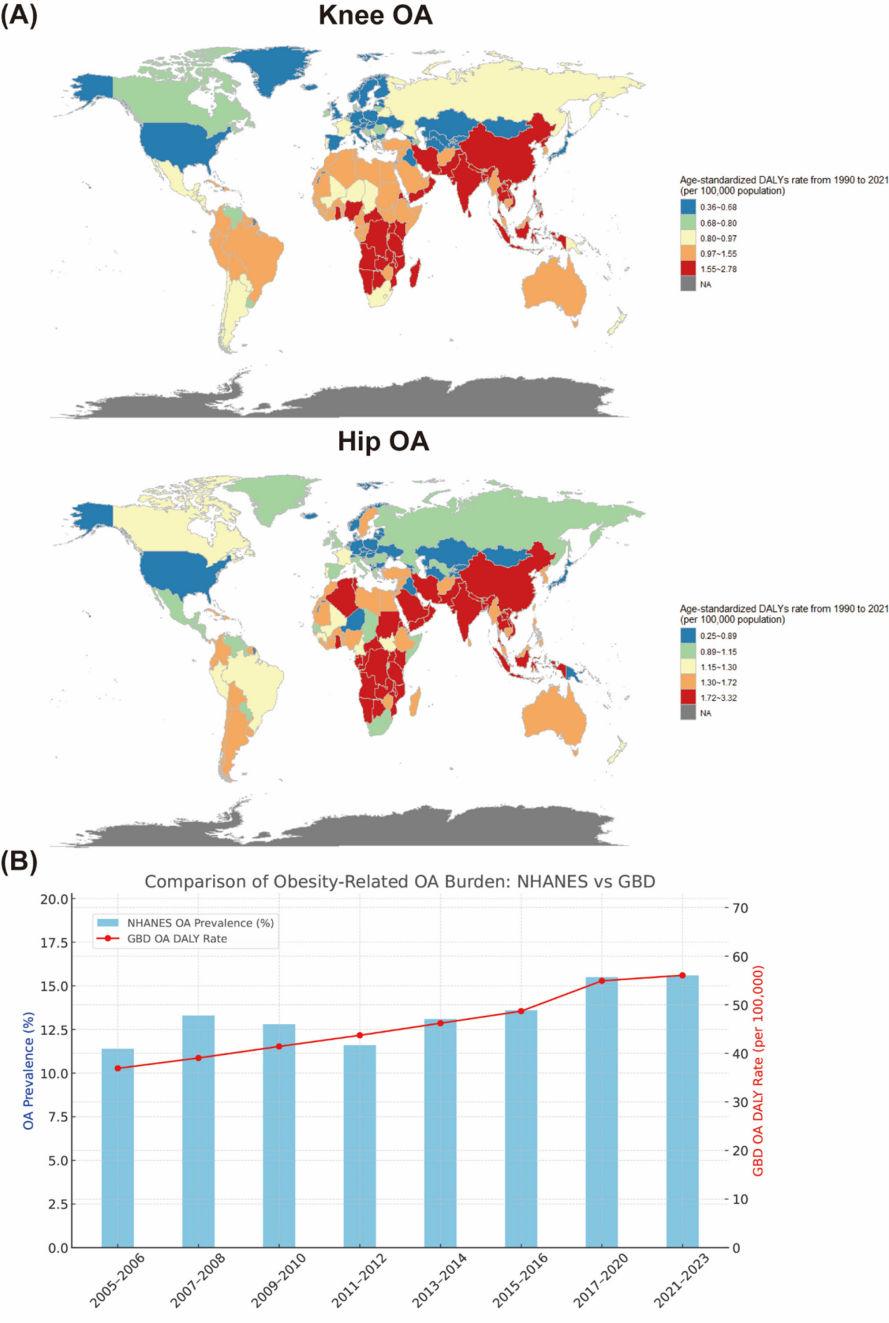
(A) Global distribution of age-standardized DALY rates in 2021 attributable to high BMI for patients with knee and hip OA. (B) Trends in obesity-related OA burden in the United States exhibiting comparison between the NHANES and GBD estimates (2005-2023). OA: Osteoarthritis.

Analysis of GBD data indicates that statistically significant increase in the high-BMI-attributable DALY burden for both knee OA and hip OA were noted across nearly all countries and territories. For knee OA, average annual percent changes (AAPCs) ranged from relatively low increase rates in countries such as Georgia (AAPC: 0.36%) and Germany (AAPC: 0.42%) to high increase rates in nations such as Bangladesh (AAPC: 2.78%), Vietnam (AAPC: 2.51%), and India (AAPC: 2.49%). Similarly, for hip OA, AAPCs demonstrated wide variation, ranging from low increase rates in Denmark (AAPC: 0.25%) and Georgia (AAPC: 0.45%) to markedly high rates in countries such as Bangladesh (AAPC: 3.32%), India (AAPC: 2.97%), and Nepal (AAPC: 2.92%) (Figure 1A). To assess the consistency of trends between national and global data sources, we compared NHANES data and GBD data. Spearman correlation analysis revealed a strong and statistically significant association between the two trends, confirming that the burden of obesity-related OA has risen steadily over the past two decades nationally and globally (Figure 1B).

NHANES data indicate that individuals with OA were significantly older (mean age 61.4 *vs*. 46.5 years) and more likely to be female (56.1% *vs*. 46.7%). Significant differences were also observed in the ethnic distribution. The proportion of non-Hispanic white participants was markedly higher in the OA group (63.0%) than in the non-OA group (48.8%). Patients with OA also exhibited a significantly greater prevalence of numerous comorbidities, including hypertension (55.1% *vs*. 30.0%), diabetes (reported prevalence 22.0% *vs*. 9.8%), anemia (6.0% *vs*. 3.5%), congestive heart failure (5.9% *vs*. 1.7%), coronary heart disease (7.8% *vs*. 2.6%), angina (4.8% *vs*. 1.1%), heart attack history (7.1% *vs*. 2.4%), stroke history (6.8% *vs*. 2.3%), liver conditions (8.0% *vs*. 3.9%), thyroid problems (19.9% *vs*. 8.7%), and malignancy (20.0% *vs*. 8.3%). Significant differences were also observed in drinking status, educational level, and marital status, whereas smoking status did not differ significantly between the groups. Laboratory assessments revealed that patients with OA had significantly lower hematocrit, lymphocyte count, hemoglobin, mean corpuscular hemoglobin concentration (MCHC), mean platelet volume (MPV), platelet count, and red blood cell count. Conversely, these patients presented with significantly elevated levels of basophils, blood cadmium, monocyte count, blood lead, mean corpuscular hemoglobin (MCH), mean corpuscular volume (MCV), segmented neutrophils, red cell volume distribution width (RDW), and white blood cell (WBC). Poverty income ratio (PIR) and sedentary time did not differ significantly between the groups.

Finally, the OA predictive discrimination capabilities of nine ML models were evaluated. The DT model yielded a training area under the curve (AUC) of 0.770 and a test AUC of 0.748 (Supplementary Figure S1). The KNN model achieved a training AUC of 0.826 and a test AUC of 0.723 (Supplementary Figure S2). The ENET model had a training AUC of 0.795 and a test AUC of 0.772 (Supplementary Figure S3). XGBoost demonstrated a strong performance, with a training AUC of 0.828 and a test AUC of 0.777 (Supplementary Figure S4). Similarly, the LightGBM model achieved a training AUC of 0.797 and a test AUC of 0.771 (Supplementary Figure S5). The logistic regression model yielded a training AUC of 0.797 and a test AUC of 0.777 (Supplementary Figure S6). The MLP model produced a training AUC of 0.815 and a test AUC of 0.782 (Supplementary Figure S7). The RF model achieved the highest test performance with an AUC of 0.80, although its near-perfect training AUC of 0.985 indicates potential overfitting (Supplementary Figure S8). Lastly, the radial support vector machine (RSVM) model yielded a training AUC of 0.794 and a test AUC of 0.769 (Supplementary Figure S9). After model correction and decision curve analysis (DCA) evaluation, the LightGBM model that consistently performed the best was selected for further explanation.^[6]^ Seven key predictive factors (age, gender, education level, diabetes, hypertension, race and thyroid disease) were identified through multi-step feature selection. To improve the accessibility and practical utility of our OA risk prediction mode, we deployed it as an interactive web application (https://Lightgbm.shinyapps.io/LightGBM/).

In conclusion, analysis of a large national dataset using interpretable ML yielded key demographical, socioeconomic, and clinical predictors of OA risk in the heterogeneous obese population. The developing personalized tools to better identify and manage high-risk individuals with obesity, thereby contributing to the goal of mitigating the substantial OA burden in this population.

## Supplementary Information

Supplementary materials are only available at the official site of the journal (www.intern-med.com).

## Supplementary Material

Supplementary Material Details
