# Supplementary material for: Application of machine learning in risk stratification of obesity-related osteoarthritis: A study based on a nationally representative sample
Source: J Transl Int Med. 2025 Nov 25;14(1):154–7. doi: 10.1515/jtim-2025-0060 (PMC12916268; doi:10.1515/jtim-2025-0060)

Figure S1: Development and evaluation of the DT model for OA prediction. (A) Hyperparameter tuning results for the DT model, showing performance metrics (Accuracy, ROC AUC, PR AUC) versus Cost-Complexity Parameter, Minimal Node Size, and Tree Depth. (B) Visualization of the final optimized DT structure. (C) ROC curves for the 5 cross-validation folds during DT training. (D) Confusion matrix for the DT model on the training dataset. (E) Confusion matrix for the DT model on the independent test dataset. (F) Final ROC curves for the optimized DT model on the training (red) and test (blue) datasets. (G) SHAP summary plot illustrating feature importance and the impact of feature values (color scale) on the DT model output. (H) SHAP dependence plots showing the relationship between individual feature values and their corresponding SHAP values for the DT model. DT: Decision Tree; OA: osteoarthritis; ROC: receiver operating characteristic; SHAP: SHapley Additive exPlanations; AUC: area under the curve; PR: precision-recall.

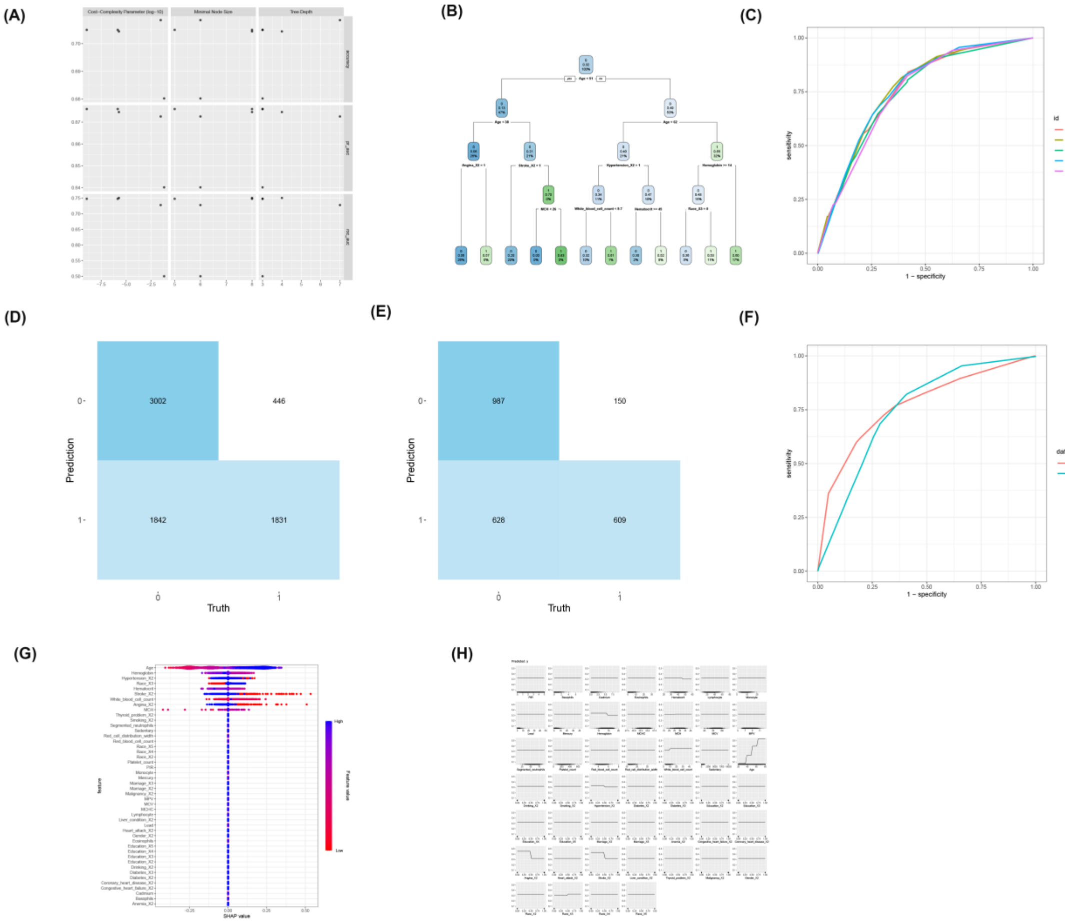

Figure S2: Development and evaluation of the KNN model for OA prediction. (A) Hyperparameter tuning results for the KNN model, showing performance metrics (Accuracy, PR AUC, ROC AUC) versus the Number of Nearest Neighbors. (B) Receiver Operating Characteristic (ROC) Area Under the Curve (AUC) across 5 cross-validation folds during KNN training. (C) ROC curves for the 5 cross-validation folds. (D) Confusion matrix for the KNN model on the training dataset. (E) Confusion matrix for the KNN model on the independent test dataset. (F) Final ROC curves for the optimized KNN model on the training (red) and test (blue) datasets. (G) SHapley Additive exPlanations (SHAP) summary plot illustrating feature importance and the impact of feature values (color scale) on the KNN model output. (H) SHAP dependence plots showing the relationship between individual feature values and their corresponding SHAP values for the KNN model. KNN: K-Nearest Neighbors; OA: osteoarthritis; ROC: receiver operating characteristic; AUC: area under the curve; SHAP: SHapley Additive exPlanations; PR, precision-recall.

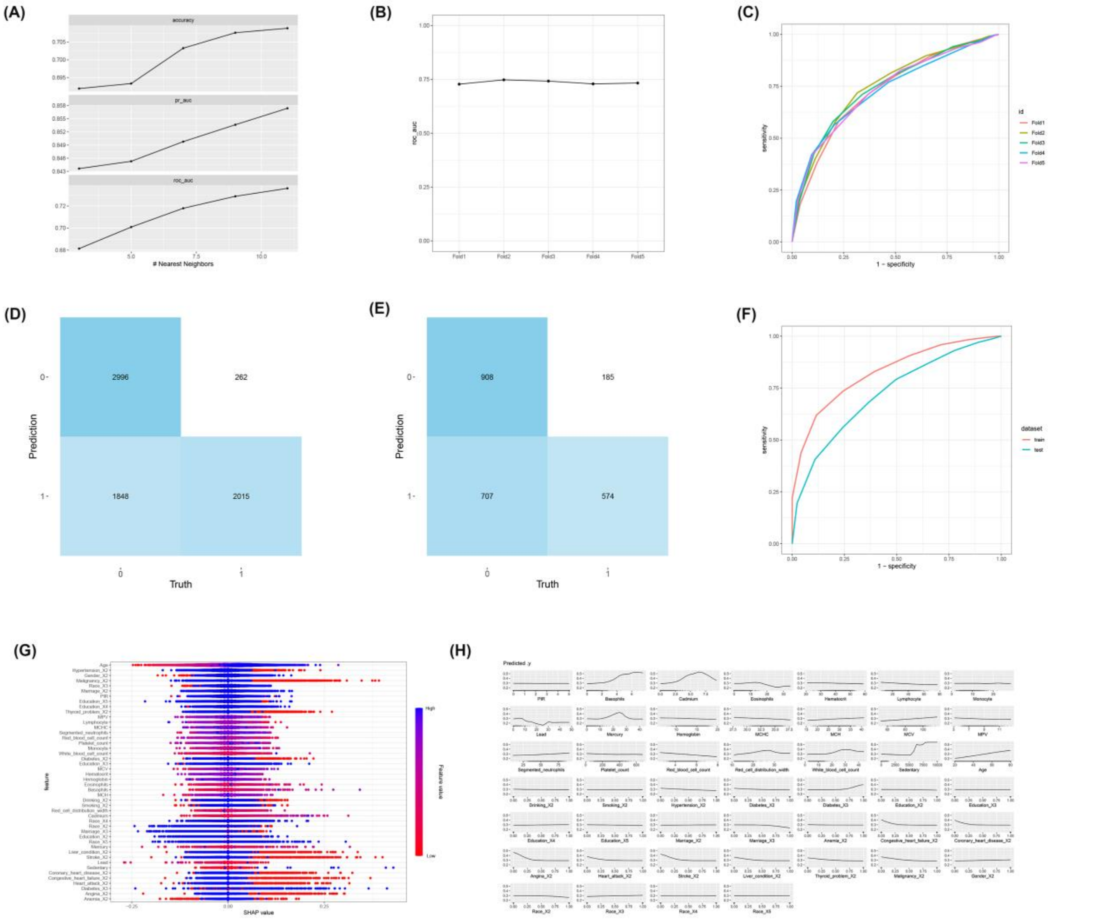

Figure S3: Development and evaluation of the ENET model for OA prediction. (A) Hyperparameter tuning results for ENET, showing performance metrics (Accuracy, PR AUC, ROC AUC) versus the Amount of Regularization and Proportion of Lasso Penalty. (B) ROC AU across 5 cross-validation folds during ENET training. (C) ROC curves for the 5 cross-validation folds. (D) Confusion matrix for the ENET model on the training dataset. (E) Confusion matrix for the ENET model on the independent test dataset. (F) Final ROC curves for the optimized ENET model on the training (red) and test (blue) datasets. (G) SHAP summary plot illustrating feature importance and the impact of feature values (color scale) on the ENET model output. (H) SHAP dependence plots showing the relationship between individual feature values and their corresponding SHAP values for the ENET model. ENET: evaluation of the Elastic Net; OA: osteoarthritis; PR: precision-recall; AUC: area under the curve; ROC: receiver operating characteristic

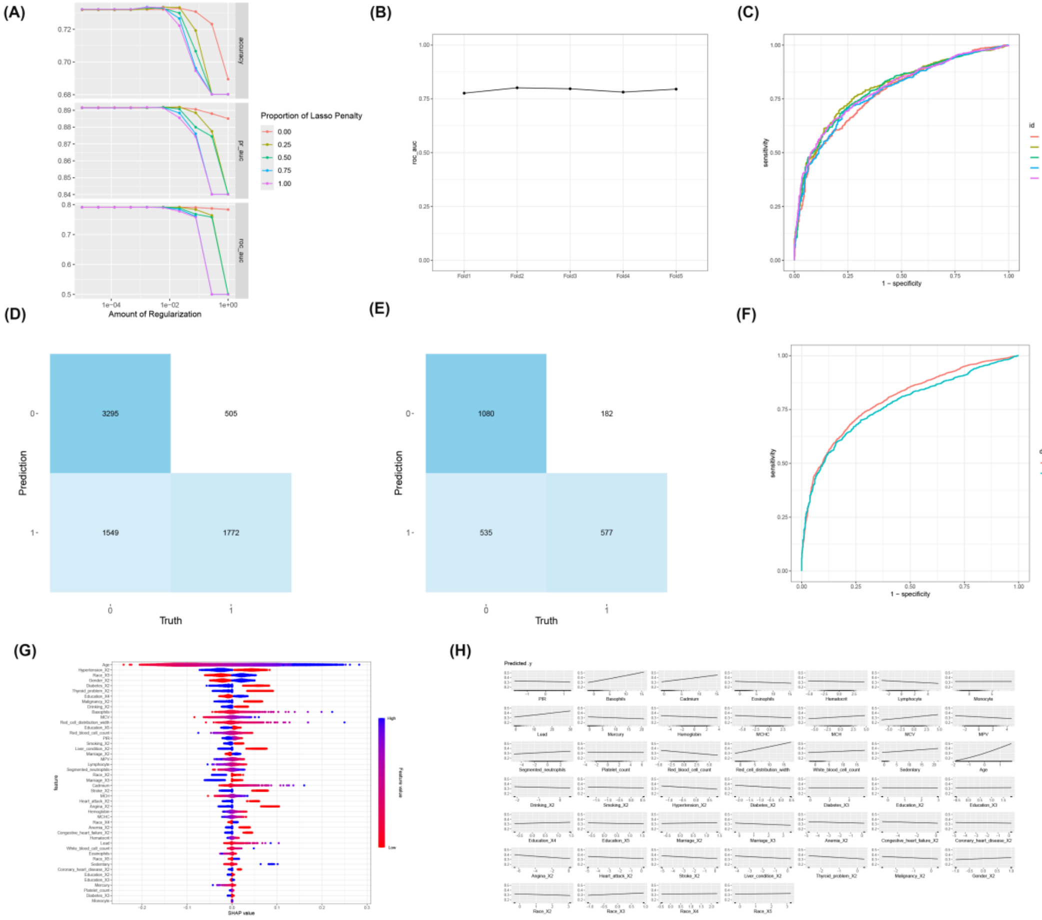

Figure S4: Development and evaluation of the XGBoost model for OA prediction. (A) Hyperparameter tuning results for XGBoost, showing performance metrics (Accuracy, PR AUC, ROC AUC) versus various hyperparameters including # Randomly Selected Predictors, # Trees, Learning Rate, Minimal Node Size, Minimal Loss Reduction, and Tree Depth. (B) ROC AUC across 5 cross-validation folds during XGBoost training. (C) ROC curves for the 5 cross-validation folds. (D) Confusion matrix for the XGBoost model on the training dataset. (E) Confusion matrix for the XGBoost model on the independent test dataset. (F) Final ROC curves for the optimized XGBoost model on the training (red) and test (blue) datasets. (G) SHAP summary plot illustrating feature importance and the impact of feature values (color scale) on the XGBoost model output. (H) SHAP dependence plots showing the relationship between individual feature values and their corresponding SHAP values for the XGBoost model. XGBoost: Extreme Gradient Boosting; OA: osteoarthritis; PR: precision-recall; AUC: area under the curve; ROC: receiver operating characteristic; SHAP: SHapley Additive exPlanations.

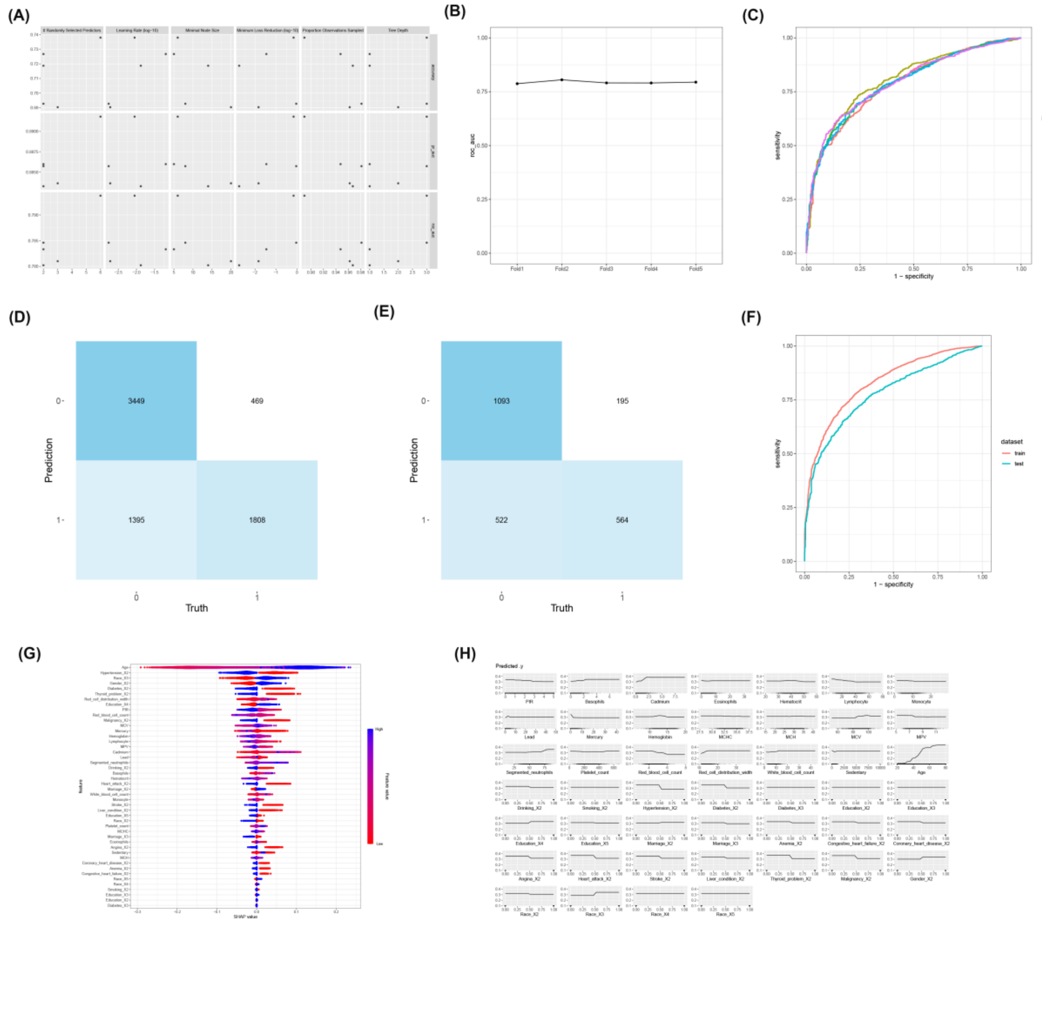

Figure S5: Development and evaluation of the LightGBM model for OA prediction. (A) Hyperparameter tuning results for LightGBM, showing performance metrics (Accuracy, PR AUC, ROC AUC versus various model hyperparameters. (B) ROC AUC across 5 cross-validation folds during LightGBM training. (C) ROC curves for the 5 cross-validation folds. (D) Confusion matrix for the LightGBM model on the training dataset. (E) Confusion matrix for the LightGBM model on the independent test dataset. (F) Final ROC curves for the optimized LightGBM model on the training (red) and test (blue) datasets. (G) SHAP summary plot illustrating feature importance and the impact of feature values (color scale) on the LightGBM model output. (H) SHAP dependence plots showing the relationship between individual feature values and their corresponding SHAP values for the LightGBM model. LightGBM: light gradient boosting machine; OA: osteoarthritis; PR: precision-recall; AUC: area under the curve; ROC: receiver operating characteristic; SHAP: SHapley Additive exPlanations.

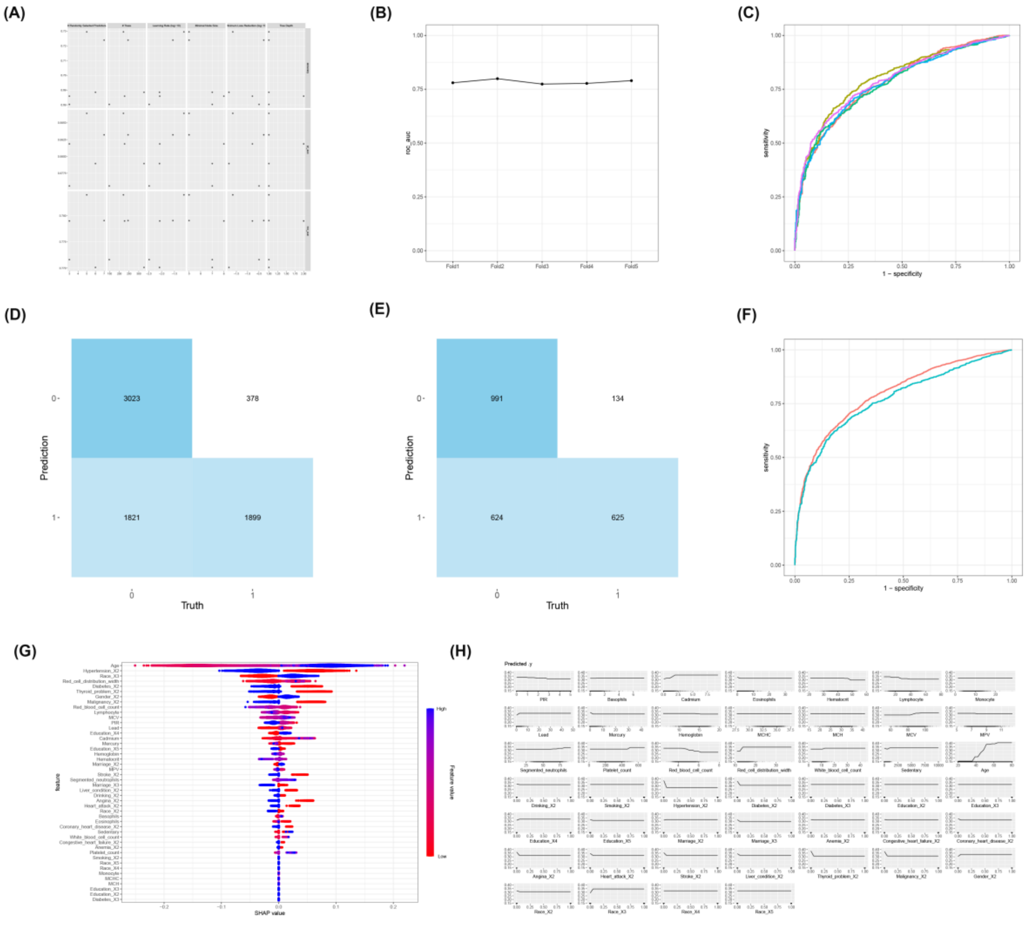

Figure S6: Development and evaluation of the Logistic Regression model for OA prediction. (A) Feature importance ranking based on the magnitude of the model coefficients (or loss function change; clarify based on plot method). (B) ROC AUC across 5 cross-validation folds during Logistic Regression training. (C) ROC curves for the 5 cross-validation folds. (D) Confusion matrix for the Logistic Regression model on the training dataset. (E) Confusion matrix for the Logistic Regression model on the independent test dataset. (F) Final ROC curves for the optimized Logistic Regression model on the training (red) and test (blue) datasets. (G) SHAP summary plot illustrating feature importance and the impact of feature values (color scale) on the Logistic Regression model output. (H) SHAP dependence plots showing the relationship between individual feature values and their corresponding SHAP values for the Logistic Regression model. OA: osteoarthritis; ROC: receiver operating characteristic; AUC: area under the curve; SHAP: SHapley Additive exPlanations.

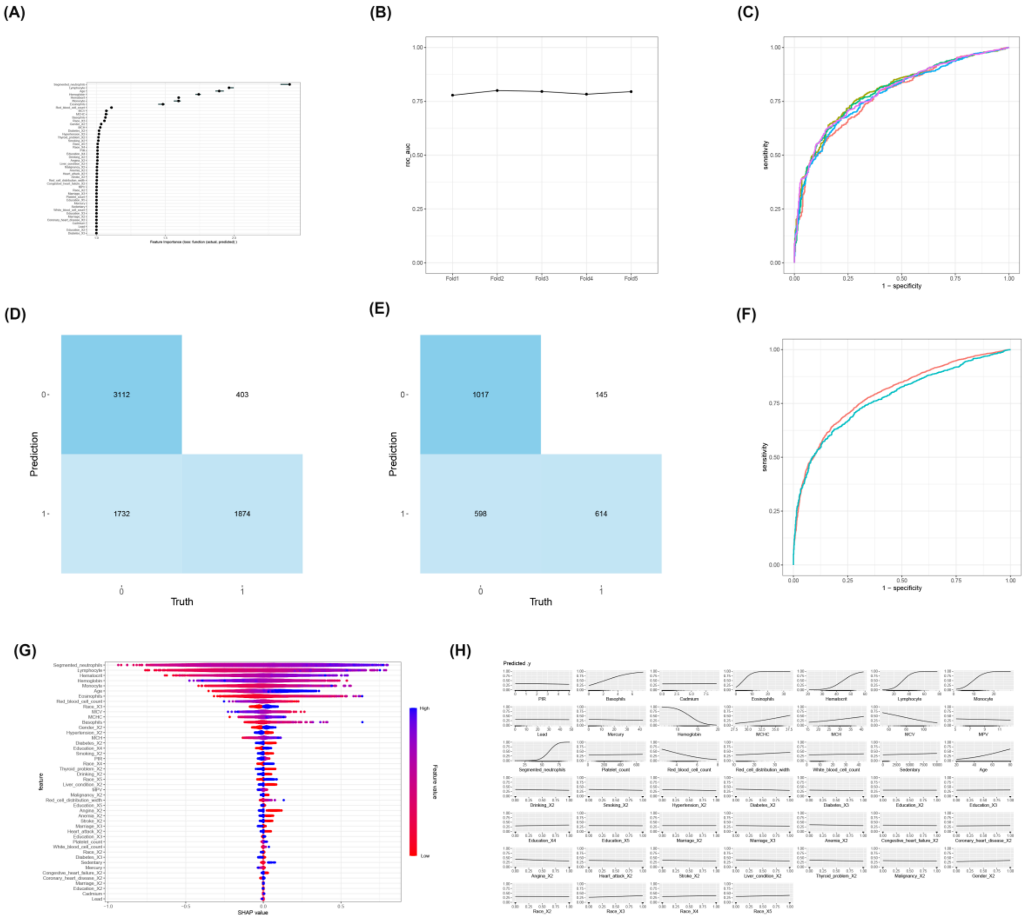

Figure S7: Development and evaluation of the MLP model for OA prediction. (A) Hyperparameter tuning results for the MLP model, showing performance metrics (Accuracy, PR AUC, ROC AUC) versus the Number of Hidden Units, Number of Epochs, and Amount of Regularization. (B) ROC AUC across 5 cross-validation folds during MLP training. (C) ROC curves for the 5 cross-validation folds. (D) Confusion matrix for the MLP model on the training dataset. (E) Confusion matrix for the MLP model on the independent test dataset. (F) Final ROC curves for the optimized MLP model on the training (red) and test (blue) datasets. (G) SHAP summary plot illustrating feature importance and the impact of feature values (color scale) on the MLP model output. (H) SHAP dependence plots showing the relationship between individual feature values and their corresponding SHAP values for the MLP model. MLP: multi-layer perceptron; OA: osteoarthritis; PR: precision-recall; AUC: area under the curve; ROC: receiver operating characteristic; SHAP: SHapley Additive exPlanations.

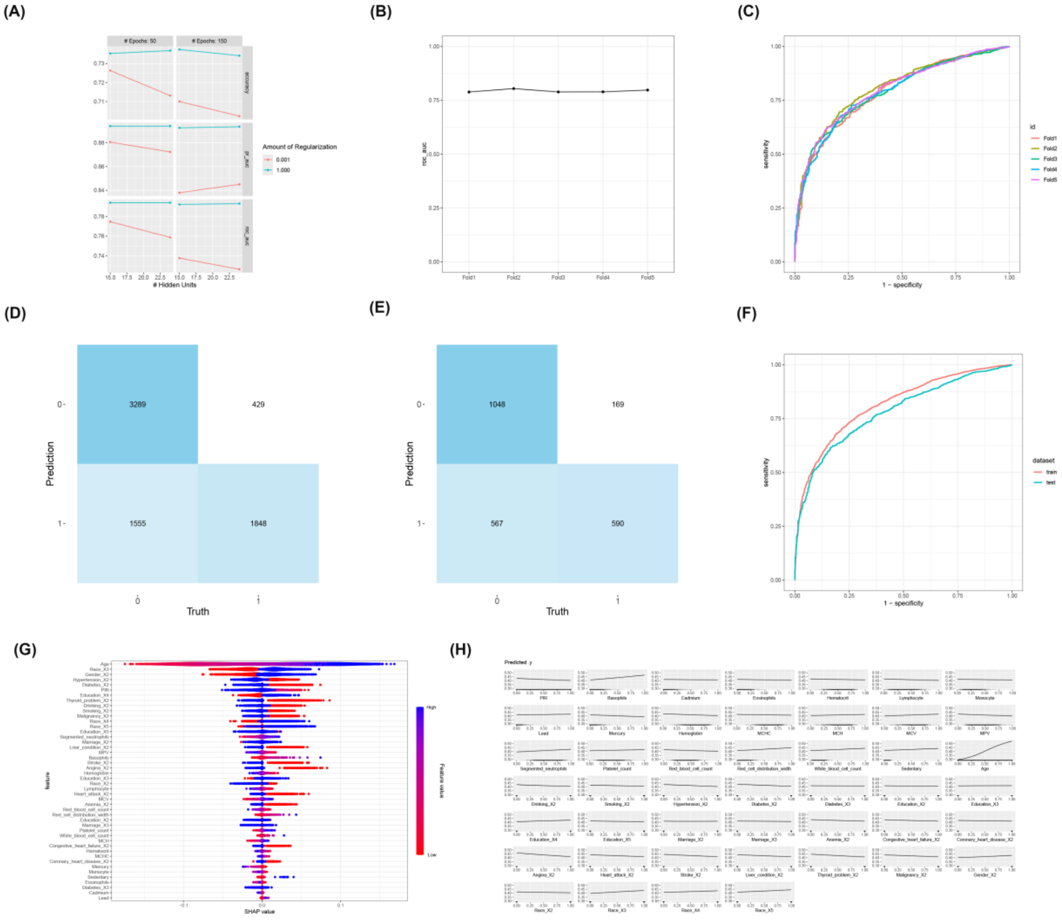

Figure S8: Development and evaluation of the RF model for OA prediction. (A) Hyperparameter tuning results for the RF model, showing performance metrics (Accuracy, PR AUC, ROC AUC) versus the Number of Randomly Selected Predictors, Number of Trees, and Minimal Node Size. (B) Error evolution during RF training, showing OOB error and class-specific errors versus the number of trees grown. (C) ROC curves for the 5 cross-validation folds during RF training. (D) Confusion matrix for the RF model on the training dataset. (E) Confusion matrix for the RF model on the independent test dataset. (F) Final ROC curves for the optimized RF model on the training (red) and test (blue) datasets. (G) SHAP summary plot illustrating feature importance and the impact of feature values (color scale) on the RF model output. (H) SHAP dependence plots showing the relationship between individual feature values and their corresponding SHAP values for the RF model. RF: random forest; OA: osteoarthritis; PR: precision-recall; AUC: area under the curve; ROC: receiver operating characteristic; OOB: out-of-bag; SHAP: SHapley Additive exPlanations.

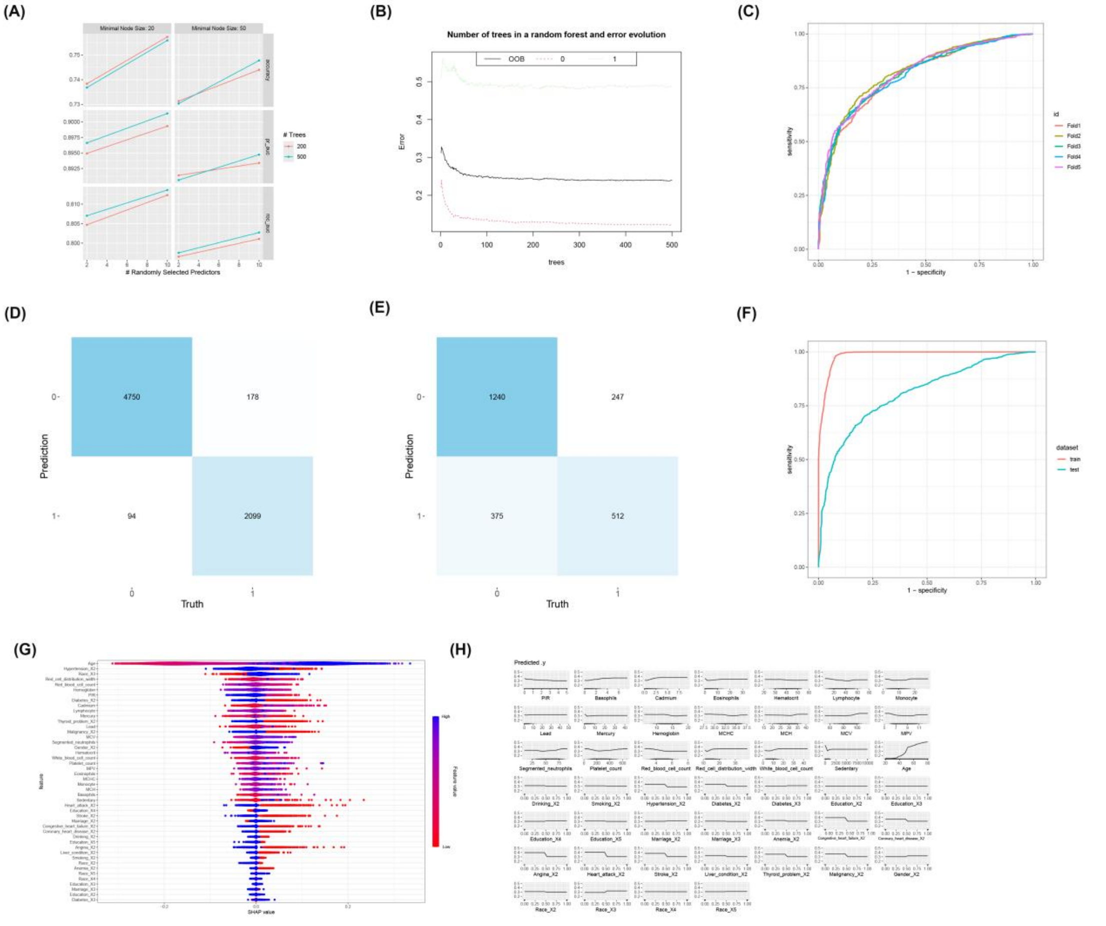

Figure S9: Development and evaluation of the SVM model with RBF kernel for OA prediction. (A) Hyperparameter tuning results for the SVM model, showing performance metrics (Accuracy, PR AUC, ROC AUC) versus the Radial Basis Function sigma and Cost parameters. (B) ROC AUC across 5 cross-validation folds during SVM training. (C) ROC curves for the 5 cross-validation folds. (D) Confusion matrix for the SVM model on the training dataset. (E) Confusion matrix for the SVM model on the independent test dataset. (F) Final ROC curves for the optimized SVM model on the training (red) and test (blue) datasets. (G) SHAP summary plot illustrating feature importance and the impact of feature values (color scale) on the SVM model output. (H) SHAP dependence plots showing the relationship between individual feature values and their corresponding SHAP values for the SVM model. SVM: support vector machine; RBF: radial basis function; OA: osteoarthritis; PR: precision-recall; AUC: area under the curve; ROC: receiver operating characteristic; SHAP: SHapley Additive exPlanations.

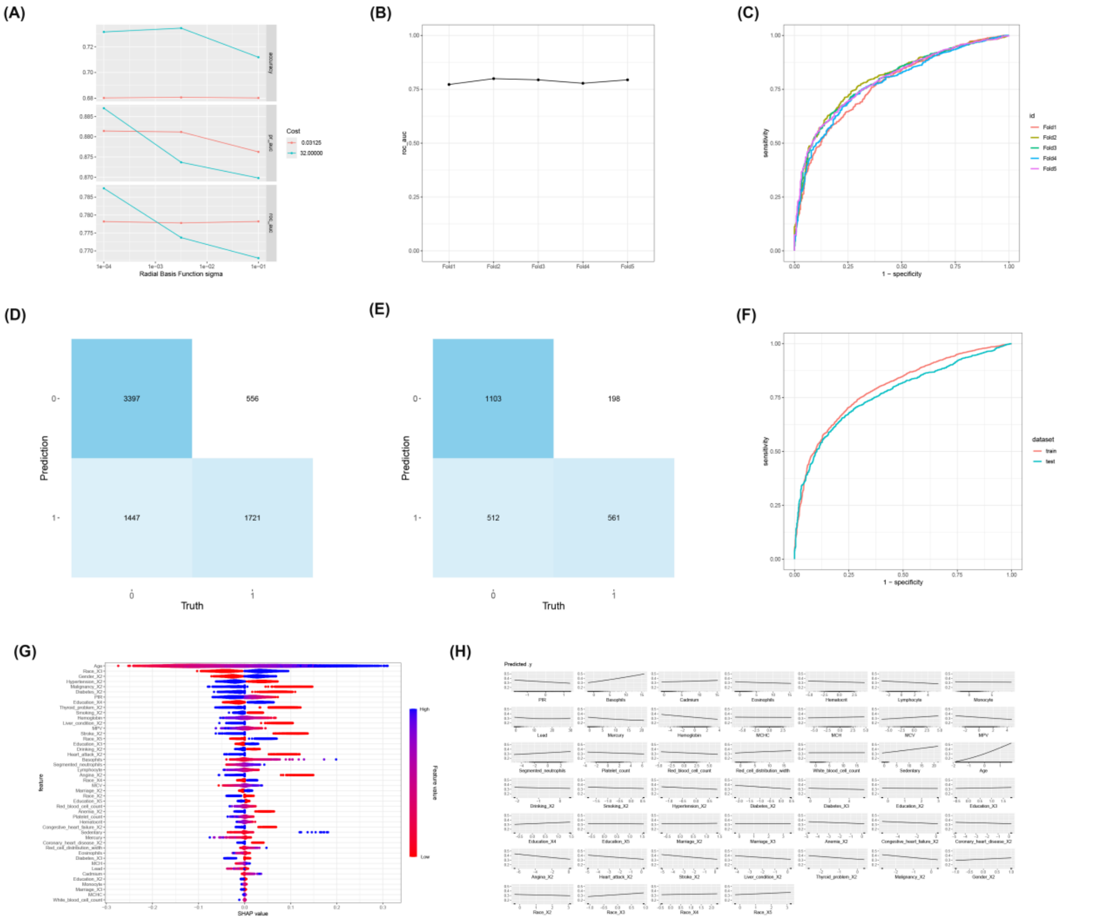

Supplement: Supplementary file 1 — Supplementary Material Details [file jtim-2025-0060_sm.pdf]
